# Serratia Marcescens outbreak on a general pediatric ward in Benin

**DOI:** 10.1186/1753-6561-5-S6-P299

**Published:** 2011-06-29

**Authors:** TA Ahoyo, MF Adeoti, AG Attolou, M Boco, LS Baba Moussa

**Affiliations:** 1 GBH Epac, University of Abomey Calavi Benin Ouest Africa , Cotonou, Benin; 2 BIOCHIMY, RIPAQS International, Abidjan, Cote d'Ivoire; 3 Quality Assurance, Hospital, Abomey, Benin; 4 Microbiology, CHDZ/C Hospital, Abomey, Benin; 5 FAST, University of Abomey Calavi Benin Ouest Africa, Cotonou, Benin

## Introduction

The consequences of infections with *Serratia marcescens *can be severe, so strategies for prevention are important.

## Objective

Our study aimed to examine the resistance in this bacterium and the main factors increasing the risk of cross contamination.

## Methods

6-months surveillance was conducted in the pediatric unit in Benin, from June 15^th^ to December 15^th^, 2009.We examined various specimenobtained from hospitalized children and 940 samples taken from hands of medical personnel, and various hospital surfaces for *Serratia marcescens* presence. Susceptibilities against antimicrobial agents were tested by the disk diffusion method according to NCCLS guidelines.Aggressive infection control measures were instituted.

## Results

324/790 (41%) patients were studied; 123 (39%) were infected by*Serratia marcescens*, including septicaemia 65 (52,8%). 121 children were colonized 1 month later after admission. From hospital environment, 108/940 (11,50%) isolates were obtained. Antimicrobial susceptibility testing revealed 56 % strains displaying multiresistance. Comparison of resistance patterns in isolates blood cultures with those from hands of personnel showed similitude in 92% of cases. Infected patients were cohorted and placed on contact precautions. Investigation by the infection control team revealed that the distributors of antiseptic were the main path of *Serratia marcescens* dissemination.

## Conclusion

New infection control policies and engineering plans were initiated on the basis of our results. Antimicrobial resistance is particularly harmful to infectious disease management in low-income countries since expensive second-line drugs are not readily available.

## Disclosure of interest

None declared.

## Note

Also presented as P349.

